# Iron Status and Systemic Inflammation, but Not Gut Inflammation, Strongly Predict Gender-Specific Concentrations of Serum Hepcidin in Infants in Rural Kenya

**DOI:** 10.1371/journal.pone.0057513

**Published:** 2013-02-27

**Authors:** Tanja Jaeggi, Diego Moretti, Jane Kvalsvig, Penny A. Holding, Harold Tjalsma, Guus A. M. Kortman, Irma Joosten, Alice Mwangi, Michael B. Zimmermann

**Affiliations:** 1 Institute of Food, Nutrition and Health, ETH Zurich, Zurich, Switzerland; 2 University of KwaZulu-Natal, Durban, South Africa; 3 International Centre for Behavioural Studies, Mombasa, Kenya; 4 Department of Laboratory Medicine, Radboud University Medical Center, Nijmegen, The Netherlands; 5 Hepcidinanalysis.com, Nijmegen, The Netherlands; 6 Department of Food, Technology and Nutrition, University of Nairobi, Nairobi, Kenya; Centro di Riferimento Oncologico, IRCCS National Cancer Institute, Italy

## Abstract

Hepcidin regulation by competing stimuli such as infection and iron deficiency has not been studied in infants and it’s yet unknown whether hepcidin regulatory pathways are fully functional in infants. In this cross-sectional study including 339 Kenyan infants aged 6.0±1.1 months (mean±SD), we assessed serum hepcidin-25, biomarkers of iron status and inflammation, and fecal calprotectin. Prevalence of inflammation, anemia, and iron deficiency was 31%, 71%, 26%, respectively. Geometric mean (±SD) serum hepcidin was 6.0 (±3.4) ng/mL, and was significantly lower in males than females. Inflammation (C-reactive protein and interleukin-6) and iron status (serum ferritin, zinc protoporphyrin and soluble transferrin receptor) were significant predictors of serum hepcidin, explaining nearly 60% of its variance. There were small, but significant differences in serum hepcidin comparing iron deficient anemic (IDA) infants without inflammation to iron-deficient anemic infants with inflammation (1.2 (±4.9) vs. 3.4 (±4.9) ng/mL; P<0.001). Fecal calprotectin correlated with blood/mucus in the stool but not with hepcidin. Similarly, the gut-linked cytokines IL-12 and IL-17 did not correlate with hepcidin. We conclude that hepcidin regulatory pathways are already functional in infancy, but serum hepcidin alone may not clearly discriminate between iron-deficient anemic infants with and without infection. We propose gender-specific reference values for serum hepcidin in iron-replete infants without inflammation.

## Introduction

Hepcidin, a 25-amino acid peptide produced and secreted mainly by hepatocytes, is a major regulator of systemic iron homeostasis [1], [2]. Hepcidin regulates iron efflux from macrophages and enterocytes through its binding to ferroportin and subsequent internalization of the receptor-ligand complex [3], [4]. It may also reduce intestinal iron absorption through ubiquitin-dependent proteasome degradation of divalent metal transporter 1 (DMT1) on the luminal enterocyte membrane [5].

Serum hepcidin transcription is decreased in iron deficiency, hypoxia and by erythropoietic stimuli, and it is increased in infection, inflammation and iron overload [2], [6]–[8]. Iron status regulates hepcidin expression primarily through the bone morphogenic protein/hemojuvelin (BMP/HJV) pathway, while infection and inflammatory cytokines such as interleukin-6 (IL-6) increase hepcidin transcription mainly through the Janus kinase/signal transducers and activators of transcription (JAK/STAT) pathway [9], [10].

The regulation of circulating hepcidin by concurrent and competing stimuli such as infection and iron deficiency has not been extensively studied in humans. Infants, particularly in low-income countries, are highly vulnerable to both, serious infections and iron deficiency [11]. Hepcidin may be a sensitive marker for iron utilization and absorption, but little data is available on hepcidin concentration and its relationship to established markers in population studies. A previous study in anemic Tanzanian children, aged 2 months to 13 years, showed high concentrations of urine hepcidin were associated with malaria, which could contribute to malarial anemia and an impaired erythropoietic response to iron supplementation [12]. In addition it was shown, that asymptomatic malarial parasitemia was associated with increased serum hepcidin concentrations and anemia in Indonesian schoolchildren, aged 5–15 years [13]. Moreover, a recent study in anemic Gambian children, aged 1.5–3 years, showed, that serum hepcidin was the major predictor of erythrocyte iron incorporation [14]. Together, these studies advocate a low-cost hepcidin assay to improve safety and efficacy of iron supplementation programs for children in developing countries. However, no published data are yet available on how inflammation and iron status interact to determine serum hepcidin concentrations in very young infants at the age of 6 month. As immune responses and hepatic metabolism are not fully mature in early infancy, hepcidin regulatory pathways may not yet be fully functional. Moreover, although reference values for serum hepcidin have been proposed for adults [15], there are no published data on serum hepcidin concentrations in healthy, iron sufficient, non-anemic infants.

Therefore, the aims of this study were to (a) investigate how iron status and inflammation interact to predict serum hepcidin concentrations in African infants, aged 6 month, in a low-income rural setting; and (b) propose a reference values for serum hepcidin in healthy, term, iron sufficient African infants.

## Methods

### Ethics Statement

Ethical approval was given by committees at the Kenyatta National Hospital/University of Nairobi (KNH-ERC/A/337), the University of KwaZulu-Natal (BF121/08), and the Swiss Federal Institute of Technology Zurich (EK 2009-N-53). Written informed consent was obtained from the care-givers. The trial was registered at clinicaltrials.gov (NCT01111864).

### Study Population and Design

We conducted a cross-sectional study as part of an iron intervention trial in Kenyan infants. From February 2010 to August 2011, we recruited 5–7 months old infants (n = 339) in the rural Msambweni district of southern coastal Kenya. The study period included two long rainy seasons from April to July (2010 and 2011) and a short rainy season from October to November 2010. Most families in this area live from subsistence farming with maize as the staple crop.

Malaria, fever, and diarrhea morbidity over the previous 3 months was assessed by questionnaire from mother’s recall, with a focus on the previous 1 week and on the day of sample collection (confirmed by health personnel). Feeding history (breastfeeding and complementary feeding) was assessed via mother’s recall. The antenatal card (n = 148) or mothers’ recall was used to record birth history and included mode of delivery, birth date and birth weight. Preterm birth was defined as registered birth before the 37^th^ week of gestation. Infant weight was measured to the nearest 100 g using a hanging scale (Salter model number 235-6S, 25 kg×100 g; Salter Brecknell, UK) and height to the nearest 0.1 cm using a measurement board (Shorr Production, LLC., Olney, MD). A whole blood sample was taken by venipuncture in the infants and a finger prick was done to measure the hemoglobin in their mothers. In a subgroup of infants (n = 148) we collected a stool sample.

### Laboratory Methods

Hemoglobin (Hb) was measured by using a HemoCue (HemoCue AB, Ängelholm, Sweden) or a HemoControl device (EKF diagnostics Sales GmbH, Barleben/Magdeburg, Germany) from a venous blood sample (3 mL) in the infants or a finger prick in their mothers. Serum was separated by centrifugation on collection day. The remaining erythrocytes were washed 3 times with normal saline and the zinc protoporphyrin to heme ratio (ZPP) was measured by using a calibrated AVIV hematofluorometer (AVIV Biomedical, Lakewood, USA). Serum ferritin (SF), soluble transferrin receptor (sTfR) and C-reactive protein (CRP) were measured at Lancet Laboratories Nairobi by using the Cobas Integra (Roche, Basel, Switzerland). The lower limit of detection (LLOD) for CRP was 0.9 mg/L. The following cut-offs were used: (a) anemia: Hb <110 g/L for infants and <120 g/L for adult women [16], (b) iron deficiency: SF <12 ng/mL [16] or sTfR ≥7.4 mg/L [17], and (c) elevated CRP: ≥4.1 mg/L (manufacturer’s reference range).

Frozen serum was transported to the Netherlands (Radboud University Medical Centre, Nijmegen) and serum hepcidin measurements were performed by a combination of weak cation exchange chromatography and time-of-flight mass spectrometry (WCX-TOF MS) [18]. An internal standard (synthetic hepcidin-24; Peptide International Inc.) was used for quantification [19]. Peptide spectra were generated on a Microflex LT matrix-enhanced laser desorption/ionisation TOF MS platform (Bruker Daltonics). Serum hepcidin-25 concentrations were expressed as nM and calculated to ng/mL (1 nM = 2.789 ng/mL). The LLOD of this method was 0.5 nM or 1.4 ng/mL, respectively. For comparison of WCX-TOF MS generated data with a previously used enzyme-linked immune-sorbent assay (ELISA) [15], hepcidin values were recalculated using the equation (ELISA-1.00)/1.52 = WCX-TOF MS determined by Kroot et al. [18].

Serum levels of human IL-1β, IL-2, IL-4, IL-5, IL-6, IL-8, IL-10, IFNγ, TNFα, and GM-CSF were determined in 156 infants by using a human cytokine multiplex kit (Cytokine 10-plex panel, Invitrogen, Breda, Netherlands) according to the manufacturer’s instructions. The LLOD of these cytokine assays was 15 pg/mL, 10 pg/mL, 5 pg/mL, 3 pg/mL, 3 pg/mL, 10 pg/mL, 5 pg/mL, 15 pg/mL, 5 pg/mL, 10 pg/mL, respectively. Serum levels of human IL-12 (p40/p70) and IL-17 were determined in 39 samples by using Singleplex bead kits (Invitrogen), also according to the manufacturer’s instructions. The LLODs were 15 pg/mL and 20 pg/mL, respectively.

Stool samples were stored at −20°C until analysis. Fecal calprotectin, a marker for intestinal inflammation, was measured in 148 stool samples using an ELISA (Calprest, Eurospital, Trieste, Italy).

### Statistical Analysis

All data were analyzed using IBM SPSS Statistics 20.0.0 (SPSS Inc., Chicago, IL) and Microsoft Office EXCEL 2010 (Microsoft, Redmond, WA). Values for samples that were below the LLOD for serum hepcidin, CRP and the cytokines were imputed using randomly generated numbers between 0 and the specific LLODs. Weight-for-age (WAZ), height-for-age (HAZ), weight-for-height (WHZ), and BMI-for age (BAZ) z-scores were calculated using the WHO Anthro software (version 3.2.2) and standards. Definition for stunting was a HAZ<-2 and for wasting a WHZ<-2 [20].

The distribution of the data was checked for normality and log transformation was performed, if needed, on non-normally distributed data and for the calculation of the geometric means (GM). Spearman correlation coefficients (rho) were determined to assess the relationship of serum hepcidin with iron status, inflammation and morbidities. Independent samples t-test and the Mann-Whitney U were used to compare serum hepcidin, iron status indices, and CRP values among groups. To explore associations between hepcidin, iron status, and inflammation, linear regression models were fitted with hepcidin, Hb, SF, sTfR, ZPP as the dependent variables. In iron replete (SF ≥12 ng/mL and sTfR <7.4 mg/L), non-anemic (Hb ≥110 g/L), full-term infants without inflammation (elevated CRP), reference values for serum hepcidin were calculated and presented as geometric means (GM) and the 2.5 and 97.5 percentiles (P2.5 and P97.5).

## Results

### Study Population

The sample included 339 mother-infant pairs. Mean (±SD) maternal age was 26 (±6.4) years, body mass index (BMI) was 21.3 (±3.1) kg/m^2^, and Hb was 116.9 (±11.7) g/L; nearly half (49.2%) of the women were anemic. Among the mothers, 95.9% were breastfeeding (n = 325) and 86.4% (n = 293) were giving complementary foods, predominantly sweetened maize gruel (“uji”), starting from four months post-delivery. Of the infants, 61.9% were born at home, 33.9% in hospital; 3.5% were preterm and 1.8% were delivered by Caesarian section. Mean (±SD) age of the infants at assessment was 6.0 (±1.1) months, and mean weight-for-age and height-for-age z-scores were −0.4 and −0.8, respectively; 4.1% of infants were wasted and 14.7% were stunted. The prevalence of anemia, iron deficiency and IDA was high: 70.5%, 25.4% and 22.4%, respectively (**Table 1**).

**Table 1:** Characteristics of the study population.

Variable	Male	Female	Both
**N**	173 (51.0%)	166 (49.0%)	339
**Age, months** *	6.0 (1.1)	6.1 (1.1)	6.0 (1.1)
**Hemoglobin, g/L** *	102.2 (11.8)	103.6 (11.1)	102.9 (10.1)
**ZPP, µmol/mol heme** *	99.6 (1.8)	84.6 (1.8)	91.9 (1.8)
**Serum ferritin, ng/mL** *			
**All children**	24.1 (2.5)	32.2 (2.7)	27.8 (2.6)
**Children without inflammation** †	21.5 (2.5)	29.5 (2.6)	24.9 (2.6)
**Soluble transferrin receptor, mg/L** *	5.8 (1.4)	5.4 (1.4)	5.6 (1.4)
**C-reactive protein, mg/L** *	1.7 (4.8)	2.3 (4.4)	2.0 (4.6)
**Hepcidin, ng/mL** * ‡	4.9 (3.5)	7.2 (3.3)	6.0 (3.4)
**IL-6, pg/mL** *	11.9 (1.9)	11.7 (1.9)	11.8 (1.9)
**Fecal calprotectin, mg/kg** *	160.7 (2.1)	175.5 (2.2)	167.1 (2.1)
**Inflammation** †	50 (28.9%)	57 (34.3%)	103 (30.7%)
**Anemia** §	132 (76.3%)	107 (64.5%)	239 (70.5%)
**Iron deficiency (ID)** ^ | |^	51 (29.8%)	35 (21.3%)	86 (25.7%)
**SF <12 ng/mL**	31 (18.3%)	22 (13.6%)	53 (15.6%)
**sTfR ≥7.4 mg/L**	36 (20.5%)	23 (14.0%)	59 (17.4%)
**Iron deficiency anemia (IDA)** ¶	50 (29.2%)	29 (17.7%)	79 (23.6%)
**Birth weight, kg**	3.2 (1.2)	3.1 (1.2)	3.1 (1.2)
**Height-for-age (z-score)**	−0.9 (1.2)	−0.7 (1.2)	−0.8 (1.2)
**Weight-for-age (z-score)**	−0.4 (1.2)	−0.4 (1.2)	−0.4 (1.2)
**BMI-for-age (z-score)**	0.1 (1.2)	−0.03 (1.3)	0.06 (1.3)
**Maternal hemoglobin, g/L** *	117.4 (11.5)	116.3 (12.0)	116.9 (11.7)

Estimates are mean (±SD) or number (%) unless indicated otherwise.

* geometric mean (±SD).

† CRP>4.1 mg/L.

‡ conversion factor: 1 ng/mL = 0.358 nM.

§ Hb <110 g/L.

| | SF <12 ng/mL or sTfR ≥7.4 mg/L.

¶ Concurrent ID and Anemia.

### Infectious Diseases

There was a high burden of infectious disease among the infants. Systemic inflammation (elevated CRP) was present in nearly one-third (30.7%) of infants, and 119 (35.1%) had a CRP below the LLOD of 0.9 mg/L. Based on maternal reports, 6.2% of infants were currently passing watery or loose stools, 1.5% were passing more than 4 stools a day, and 17.1% reported blood or mucus in the stools. One infant had malaria on the day of the venipuncture (assessed by a Giemsa stained blood smear), and 7.4% of infants had had malaria in the previous 3 months (reported by caretaker). Mothers reported 8% of infants had received antibiotics, 2.9% supplements for diarrhea, and 2.7% treatment for intestinal parasites (reported as “worms”) within the previous week.

### Serum Hepcidin and its Interaction with Biochemical, Anthropometric and Health Markers

**Figure 1:**
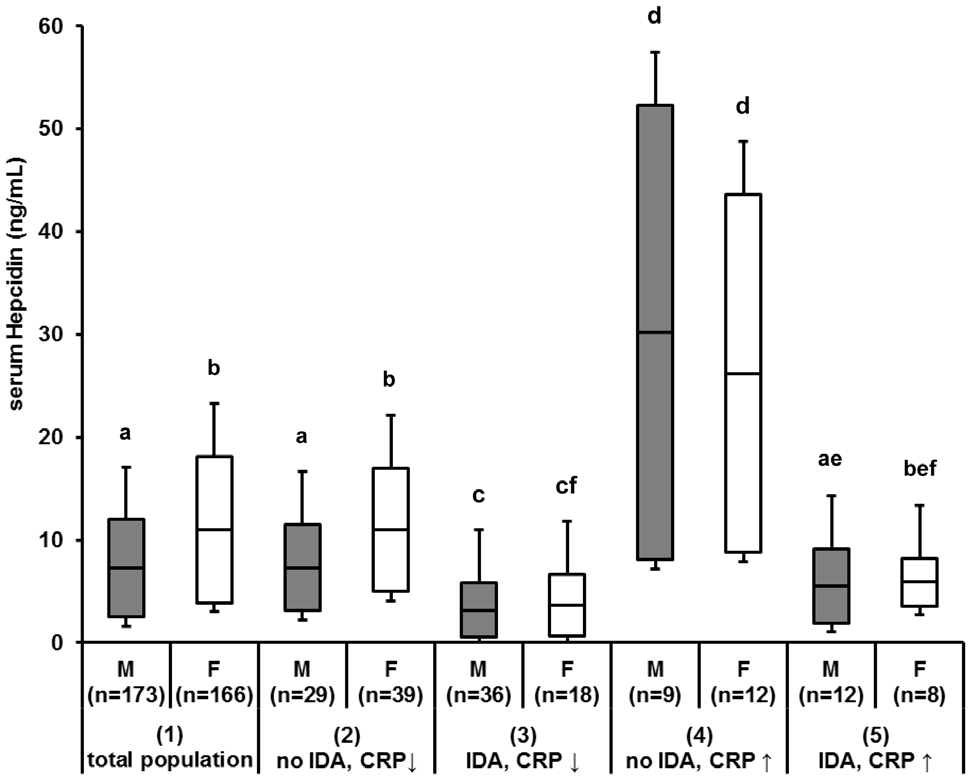
Median levels, lower and upper quartile plus standard error, of serum hepcidin (ng/mL) by sex, measured in different subgroups. Criteria for subgroups were as follows: (**1**) total population; (**2**) no iron deficiency (ID), no anemia, and no elevated CRP concentration; (**3**) ID and anemia (IDA), but no elevated CRP concentration; (**4**) no ID and anemia, but elevated CRP concentration; (**5**) IDA and elevated CRP concentration. Cut-offs were as follows: anemia: Hb <110 g/L; ID: SF <12 ng/mL or sTfR ≥7.4 mg/L; IDA: concurrent anemia and ID; no ID: SF ≥12 ng/mL and sTfR <7.4 mg/L; elevated CRP: CRP≥4.1 mg/L. Note: all individuals with ID and no elevated CRP were anemic. Letters (a, b, c, d, e, and f) indicate significant differences at P<0.05.

Among infants, geometric mean (GM, (±SD)) serum hepcidin was 6.0 (±3.4) ng/mL (**Table 1**), and was below the lower limit of detection (LLOD) in 51 (15.0%) infants. Serum hepcidin was significantly lower in males than in females (GM (±SD) 4.9 (±3.5) ng/mL vs. 7.2 (±3.3) ng/mL; P<0.05; **Figure 1**, (1) total population). Infants born preterm had significantly lower serum hepcidin (0.7 (±7.0) ng/mL (58% below LLOD) vs. 7.1 (±3.9) ng/mL (13% below LLOD); P<0.05) and higher ZPP values (143.7 (±1.9) mol/mol heme vs. 83.8 (±1.8) mol/mol heme; P<0.05) compared to full-term infants. There were no statistically significant differences in Hb, SF, TfR and CRP concentrations comparing preterm to term infants, but these comparisons were limited as only 3.5% of infants were known to be born preterm.

Serum hepcidin was significantly correlated with Hb, ZPP, SF, sTfR, CRP, IL-6, weight, height, gender and premature birth (**Table 2**). There were no significant correlations of serum hepcidin with maternal Hb, place of delivery (hospital or home) or infectious disease history. In the multiple linear regression analyses including gender and weight-for-age z-score (WAZ); CRP (β = .273), IL-6 (β = .221), ZPP (β = −.337), and SF (β = .274) were significant predictors of serum hepcidin, resulting in an adjusted R^2^ of.595 (P<0.001; **Table 3**). CRP and IL-6 together explained 22.8% of the variance in serum hepcidin (adjusted R^2^ = .228). Omitting CRP and IL-6 from the model, the remaining iron biomarkers explained 36.7% of the variance in serum hepcidin (adjusted R^2^ = .367). Replacing ZPP with sTfR (β = −.202, P<0.001) did not change the predictive power of the model. ZPP and sTfR correlated strongly (Spearman coefficient rho = .628, P<0.001; **Table 2**), and including one of these predictors in the regression model explained nearly the same variance in serum hepcidin as both together. IL-6 was the only cytokine with a significant correlation with hepcidin (rho = .358; P<0.01, **Table 2**); moreover IL-6 correlated with SF (rho = .210; P<0.01) and CRP (rho = .554; P<0.01). IL-5 correlated with Hb (rho = −.176; P<0.05), and IL-12 with SF (rho = −.364; P<0.05). Most interleukins correlated with CRP, but not with serum hepcidin (**Table 4**).

**Table 2:** Spearman correlation coefficients (rho) from iron and inflammation markers and anthropometric data of the study infants.

	ZPP	SF	sTfR	CRP	Hepcidin	IL-6	Gender	BW	Weight	Height
**Hb, g/L**	−.498*	.242*	−.431*	−.144*	.186*	−.132	.107	.107	−.135†	.009
**ZPP, µmol/mol heme**	−	−.439*	.628*	−.002	−.437*	−.001	.205†	−.170†	.205*	.010
**SF, ng/mL**	−	−	−.417*	.251*	.609*	.210*	.144*	.201†	−.195*	−.134†
**sTfR, mg/L**	−	−	−	.017	−.371*	.065	−.158*	−.161	.130†	.056
**CRP, mg/L**	−	−	−	−	.426*	.554*	.122†	.014	−.037	−.056
**Hepcidin, ng/mL**	−	−	−	−	−	.358*	.206	.131	−.232*	−.152*
**IL-6, pg/mL**	−	−	−	−	−	−	.011	.062	−.116	−.034
**Gender, (m/f)**	−	−	−	−	−	−	−	−.113	−.258*	−.243*
**BW, kg**	−	−	−	−	−	−	−	−	.213*	.135
**Weight, kg**	−	−	−	−	−	−	−	−	−	.632*

* P<0.01;

† P<0.05; BW = birth weight.

**Table 3:** Results of linear regression analyses for serum hepcidin (ng/mL) in the total population and by gender separately.

	Total population			Male			Female		
	(n = 141)			(n = 65)			(n = 76)		
	B	SE B	β	B	SE B	β	B	SE B	β
**Constant**	1.264	.274		.987	.471		1.606	.326	
**CRP, mg/L**	.168	.039	.273*	.162	.059	.288†	.168	.058	.257†
**IL**−**6, pg/mL**	.306	.088	.221*	.287	.141	.207†	.323	.122	.242†
**ZPP, µmol/mol heme**	−.574	.125	−.337*	−.390	.213	−.218	−.670	.163	−.414*
**sTfR, mg/L**	−.101	.157	−.044	−.126	.230	−.063	−.130	.246	−.050
**SF, ng/mL**	.246	.056	.274*	.267	.113	.265†	.244	.066	.297*
**WAZ**	−.052	.020	−.151†	−.056	.027	−.187†	−.051	.031	−.128
**Gender**	.076	.043	.097	−	−	−	−	−	−

Note: Adjusted R^2^ Total = .595; Adjusted R^2^ Male = .553; Adjusted R^2^ Female = .586;

* P<0.001;

† P<0.05. The dependent variable hepcidin and the independent variables were log-transformed before inclusion in the models. Interpretation for these betas is as follows: 1% change in the independent variable corresponds to a beta% change in the dependent variable.

**Table 4:** Mean levels of cytokines and fecal calprotectin and their correlation with hepcidin and CRP.

	Geometric mean (± SD)	Correlation (rho) with Hepcidin	Correlation (rho) with CRP
**IL-1, pg/mL**	18.5 (2.5)	.023	.257†
**IL-2, pg/mL**	24.0 (1.6)	.084	.261†
**IL-4, pg/mL**	12.0 (7.5)	.086	.252†
**IL-5, pg/mL**	4.5 (1.7)	.021	.159
**IL-6, pg/mL**	11.8 (1.9)	.405*	.538*
**IL-8, pg/mL**	42.5 (2.4)	.190	.291*
**IL-10, pg/mL**	28.4 (1.5)	.093	.265†
**IFN-γ, pg/mL**	3.0 (3.7)	−.034	−.052
**TNF-α, pg/mL**	9.5 (2.7)	−.040	.088
**GM-CSF, pg/mL**	8.2 (3.1)	−.002	.194
**IL-12, pg/mL**	237.3 (2.0)	.011	.179
**IL-17, pg/mL**	38.7 (2.1)	.216	.383
**Calprotectin, mg/kg**	167.1 (2.1)	−.087	−.088

* P<0.01;

† P<0.05.

The gender-specific regression models had a R^2^ of.553 and.586 in male and female infants respectively (**Table 3**). Serum ferritin was a stronger predictor of hepcidin in females (β = .297, P<0.001) than in males (β = .265, P<0.05), while CRP was a slightly stronger predictor of hepcidin in males (β = .288, P<0.001) compared to females (β = .257, P<0.001). WAZ was a significant predictor (P<0.05) for hepcidin in the total population and in female infants, but not in male infants.

**Figure 2:**
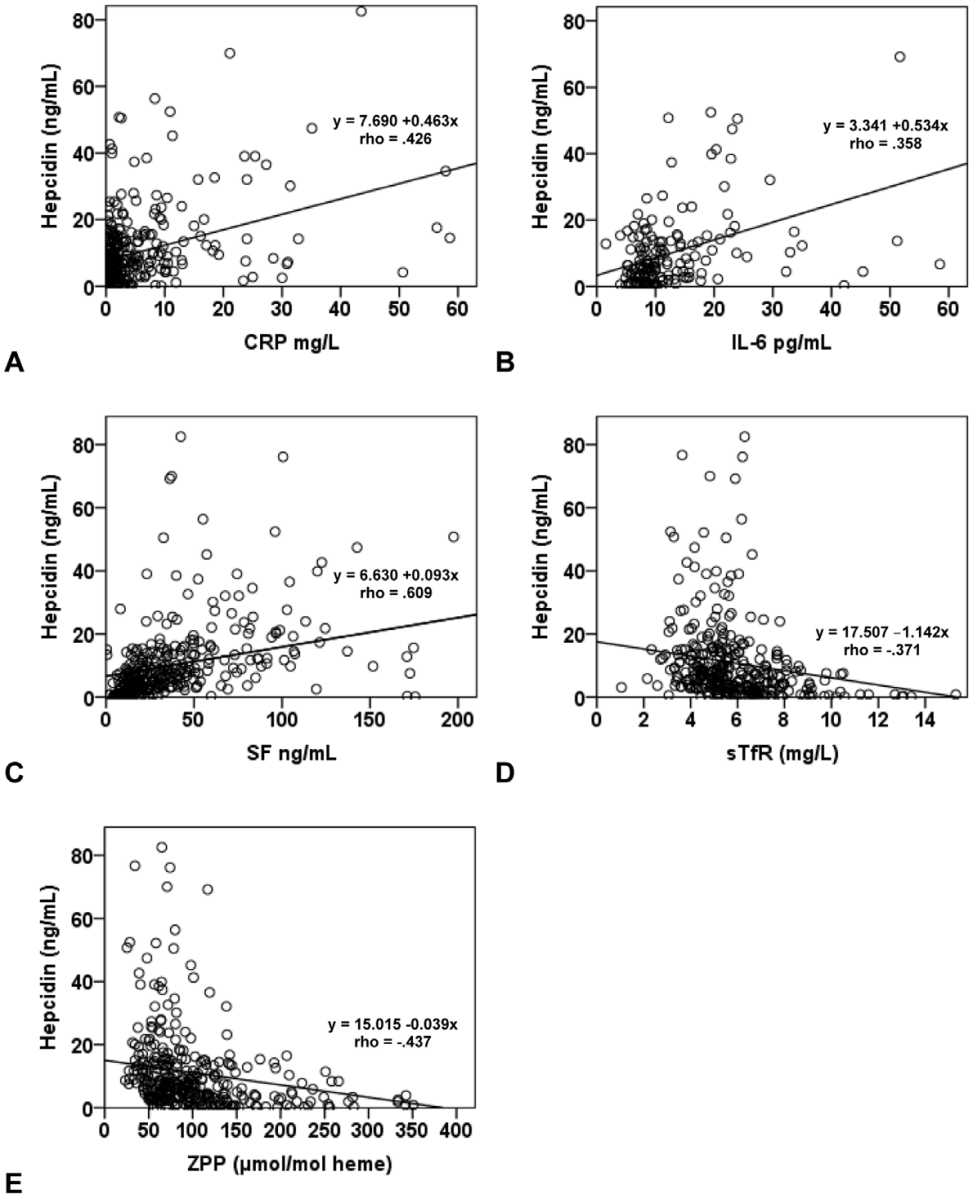
Scatter plots and regression equations derived from the correlation of inflammation markers (A: CRP and B: IL-6) and iron markers (C: SF, D: sTfR, and E: ZPP) with serum hepcidin.

CRP correlated significantly with SF (rho = .251; P<0.01), Hb (rho = −.144; P<0.01), and hepcidin (rho = .426; P<0.01), but not with sTfR and ZPP. **Figure 2** shows scatterplots of hepcidin with inflammation and iron markers. Separate linear regression analyses with the iron markers (Hb, SF, sTfR, ZPP and hepcidin) as dependent variables and inflammation markers (CRP and cytokines) as independent variables, identified CRP as a significant predictor for SF (β = .254; P<0.001), Hb (β = −.185; P = 0.001) and hepcidin (β = .394; P<0.001), but not for sTfR and ZPP. CRP and the cytokines together resulted in an R^2^ of.345 (P<0.05) for the prediction of SF concentration, whereas sTfR and ZPP were not predicted by CRP and cytokines.

### Fecal Calprotectin

Geometric mean (±SD) of fecal calprotectin (FC) was 167 (±1.0) mg/kg. Calprotectin did not correlate with any other inflammation or iron marker, but it correlated significantly with weight (rho = −.258; P<0.01) and the report of the mothers of blood or mucus in stool (rho = −.256; P<0.01). Fecal calprotectin values did not correlate with any other morbidity indices or the duration of breastfeeding. Only four mothers reported current diarrhea (reported as 4 or more loose stools a day) in their children, in 3 of them FC values are available. There was a trend for a significant correlation between diarrhea cases and FC (rho = −.158; P = 0.06). Girls had slightly higher fecal calprotectin values (GM (± SD)) than boys, of 175.5 (±1.0) mg/kg and 160.7 (±1.0) mg/kg, respectively. Fecal calprotectin did not correlate with the gut-linked cytokines IL-12 and IL-17 measured in serum, and these cytokines did not correlate with hepcidin.

### Subgroup Analysis of Serum Hepcidin According to Iron and Inflammation Status

**Figure 1** shows gender-stratified serum hepcidin concentration in different subgroups based on inflammation (measured by CRP) and iron status and anemia. Twenty-nine male and thirty-nine female infants were iron sufficient, non-anemic and had no elevated CRP. The reference values for serum hepcidin from these were 4.1 ng/mL in male and 9.0 ng/mL in female infants (**Table 5**). Serum hepcidin (GM ±SD) in iron deficient infants without elevated CRP were low (1.0 (±4.2) ng/mL and 1.5 (±3.8) ng/mL for males and females respectively); 23 out of the 36 males (63.9%) and 9 out of the 18 females (50.0%) had serum hepcidin concentration below the detection limit of 1.4 ng/mL. We found small, though significant differences in mean hepcidin concentration comparing iron deficient anemic infants without inflammation (3 in **Figure 1**) to iron deficient anemic infants with inflammation (5 in **Figure 1**; 1.2 (±4.9) ng/mL vs. 3.4 (±4.9) ng/mL; P<0.001).

**Table 5:** Reference values for serum hepcidin (nM and ng/mL) from non-anemic, iron replete, Kenyan infants without elevated CRP (CRP<4.1 mg/L AND SF ≥12 ng/mL AND sTfR <7.4 mg/L AND Hb ≥110 g/L).

			95% reference range	
		Geometric Mean	P2.5	P97.5
**Male (n = 29)**	**nM**	1.5	0.0	5.3
	**ng/mL**	4.1	0.1	14.8
**Female (n = 39)**	**nM**	3.2	0.1	18.2
	**ng/mL**	9.0	0.3	50.1
**All (n = 68)**	**nM**	2.3	0.1	18.1
	**ng/mL**	6.4	0.2	50.6

## Discussion

We are not aware of previous reports of serum hepcidin (hepcidin-25) concentrations in young full-term infants. Previous data on serum hepcidin in infancy is limited to measurements in preterm infants, low birth weight newborns in cord blood and children over the age of 18 months [13]–[15]. Pro-hepcidin concentrations in healthy non-anemic Turkish infants were reported to be 118±102.2 ng/mL (mean ± SD) [21], although it is questionable whether these reflect any physiological relevance [22], [23]. In our Kenyan male infants, hepcidin concentrations were much lower than those previously found in 18–24 years old Dutch men and adapted for the differences in methods (4.1 ng/mL vs 14.9 ng/mL) [15]. In female Kenyan infants, however, we found higher values (9.0 ng/mL vs 2.9 ng/mL) than in 18–24 years old women from the previously mentioned Dutch population. Therefore, in contrast to serum hepcidin concentrations in adults where males have generally higher values than females [15], [24], serum hepcidin in our study was found to be around 50% higher in female infants than in males, after controlling for weight and height. In our regression model including iron status (sTfR, SF and ZPP) and inflammation markers (CRP and IL-6), gender is not a significant predictor (**Table 3**). This gender difference in hepdicin concentration can mainly be explained by the lower overall iron status in males, and the higher CRP concentration in females. Several studies have reported higher rates of anemia and lower iron status in male compared to female infants [25]–[29], which were attributed to gender-specific growth rates [26] or hormone-mediated differences in iron metabolism [29]. However, more research is needed to elucidate gender-differences in iron status in infants. The percentage of exclusively breastfed infants was slightly higher in males (6.6%) compared to females (3.1%). Even if not statistically significant, the higher proportion of breastfed boys may contribute to the lower prevalence of infection in boys found in this population group; as breast milk has shown to be protective against infectious diseases [30].

This study demonstrates that circulating hepcidin-25 concentrations are strongly predicted by both infection/inflammation markers (CRP and IL-6) and iron status markers (SF, ZPP and sTfR) in infants in rural sub-Saharan Africa. These data suggest that the known adult hepcidin regulatory pathways are functioning well as early as 6 months of age. Inflammation and iron status explained nearly 60% of the variance in serum hepcidin, with a roughly equal contribution from each in this setting: CRP and IL-6 explained 23% of the variance in serum hepcidin, SF alone (a reflection of both iron status and, as an acute-phase protein, inflammation) explained 32% of the variance, while ZPP and sTfR together explained 21% (ZPP alone 20% and sTfR 12%, respectively). In a previous study, in cord blood from low birth weight newborns and preterm infants, circulating hepcidin concentration was also correlated with iron status [31], [32]. Also in Dutch adults, the strongest predictor of serum hepcidin was SF (R^2^ = .58 and R^2^ = .62 in adult men and women, respectively), followed by CRP [15], but infection/inflammation is much more common in our sample of Kenyan infants. Notably, hepcidin levels in iron deficient and anemic (IDA) children with elevated CRP were significantly lower than those in non-anemic children with elevated CRP. Although IDA children with inflammation tended to have higher hepcidin levels compared to IDA children without inflammation, this difference was significant in male infants only (**Figure 1**). These findings suggest that the inflammation mediated stimuli (through IL-6) are to a large extent overruled by iron demand and erythropoiesis stimuli down regulating hepcidin synthesis.

Defining iron status in Sub-Saharan Africa and other settings with a high burden of infectious disease is difficult [33]. The use of SF is limited by its confounding by inflammation, and ZPP and sTfR have been proposed as preferable indices in these settings, although ZPP may also be confounded by inflammation and sTfR by the rate of erythropoesis. In our infant population, CRP correlated significantly with SF but not with sTfR and ZPP. In a multiple regression model, CRP and the cytokines together explained SF with an R^2^ of.345 (P<0.005), whereas sTfR and ZPP were not predicted by CRP and the cytokines. Thus, these data suggest sTfR and ZPP are suitable iron biomarkers in this age group and setting, but standardized cut-offs for infants are needed [34]–[36].

CRP, a downstream biomarker of inflammation, was a major predictor of serum hepcidin in our infant population. IL-6 induces hepcidin expression [37], [38] and it is thought that the increase in circulating hepcidin during infection/inflammation is primarily mediated by IL-6 [23]. The inclusion of IL-6 in our regression analysis significantly increased the variance in serum hepcidin explained by the model (R^2^ = .587). CRP and IL-6 together showed an adjusted R^2^ of.228, while the adjusted R^2^ of CRP and IL-6 alone was.199 and.162, respectively. These data suggest other factors, along with IL-6, may increase CRP during inflammation; however, IL-6 was the only cytokine with a significant correlation with serum hepcidin in our models.

Fecal calprotectin (FC), a biomarker of localized gut inflammation, was correlated with the report of recent blood or mucus in the infant stool. Similarly, there was a borderline significant trend for a correlation of FC with the few cases of diarrhea reported in this population at the time of assessment. However, FC was not correlated with systemic inflammation, as measured by CRP or the cytokines. Especially, IL-12 and IL-17, which have been linked to gut inflammation [39], [40], did not show any correlation with FC. Furthermore, FC, IL-12 and IL-17 were not correlated with serum hepcidin, suggesting that gut inflammation is not a predictor for hepcidin in this population. It should however be noted that, except for IL-6, none of the other measured cytokines were correlated with hepcidin. Gut inflammation has been associated with an IL-6-mediated hepcidin increase in a mouse model for intestinal colitis, but results in humans with inflammatory bowel disease (IBD) are equivocal [41]. The young age of the Kenyan infants, which is associated with a developing gut microbiota and immature intestinal immune system [42], might play a role in the absence of hepcidin induction in the infants with increased FC levels. Higher values of FC have been reported in breastfed compared to formula fed Italian infants [43], however, in our population duration of breastfeeding did not predict FC. The younger Italian infants (2–13 weeks) showed median FC concentration of 555 mg/kg, thrice as high than in our Kenyan infant population (167.1 mg/kg) [43]. However, healthy Ugandan infants aged 0–1 years had a median FC of 249 mg/kg, similar to our study population [44].

The present study provides important data on serum hepcidin concentration in iron replete, iron deficient, and iron deficient anemic infants in rural sub-Saharan Africa. We also propose a gender-specific reference values for serum hepcidin measured by WCX-TOF MS in healthy, full-term, iron replete, non-anemic African infants. The association of hepcidin with iron status and infection markers in infancy indicates the potential relevance of hepcidin as iron status marker in this population group. Our data shows significant differences in hepcidin concentration in IDA infants with and without inflammation. However, a separate analysis of this subgroup by sex resulted in significant differences in boys, but not in girls. Furthermore the size of the differences and the relatively large standard deviations also suggest that serum hepcidin alone may not provide sufficient discriminatory power between anemic infants with and without inflammation (**Figure 1**). Thus, because provision of iron to infants with infections may be dangerous in certain settings, more research is clearly needed on the potential utility of hepcidin as a point-of-care marker to increase the safety of iron supplementation in infants and children in the developing world, as has been proposed [12], [13]. In addition, our data suggest serum hepcidin is not correlated with gut inflammation during infancy; thus, the potential local injurious effect of dietary iron supplements on infectious or inflammatory intestinal disease [45], [46] may not be predicted by serum hepcidin levels. In conclusion, although promising, our study underscores that the potential clinical advantage of serum hepcidin to guide safe iron supplementation in infancy needs further clarification.
